# Recommendations for a Better Understanding of Sex and Gender in the Neuroscience of Mental Health

**DOI:** 10.1016/j.bpsgos.2023.100283

**Published:** 2023-12-30

**Authors:** Lara Marise Wierenga, Amber Ruigrok, Eira Ranheim Aksnes, Claudia Barth, Dani Beck, Sarah Burke, Arielle Crestol, Lina van Drunen, Maria Ferrara, Liisa Ann Margaret Galea, Anne-Lise Goddings, Markus Hausmann, Inka Homanen, Ineke Klinge, Ann-Marie de Lange, Lineke Geelhoed-Ouwerkerk, Anna van der Miesen, Ricarda Proppert, Carlotta Rieble, Christian Krog Tamnes, Marieke Geerte Nynke Bos

**Affiliations:** a Institute of Psychology, Leiden University, Leiden, the Netherlands; b Leiden Institute for Brain and Cognition, Leiden University, Leiden, the Netherlands; c Division of Psychology and Mental Health, School of Health Sciences, Faculty of Biology, Medicine and Health, University of Manchester, Manchester, United Kingdom; d Autism Research Centre, Department of Psychiatry, University of Cambridge, Cambridge, United Kingdom; e Department of Psychiatric Research, Diakonhjemmet Hospital, Oslo, Norway; f NORMENT, Institute of Clinical Medicine, University of Oslo, Oslo, Norway; g PROMENTA Research Center, Department of Psychology, University of Oslo, Oslo, Norway; h Interdisciplinary Center for Psychopathology and Emotion regulation, Department of Psychiatry, University Medical Center Groningen, University of Groningen, Groningen, the Netherlands; i Department of Neuroscience and Rehabilitation, Institute of Psychiatry, University of Ferrara, Ferrara, Italy; j University Hospital Psychiatry Unit, Integrated Department of Mental Health and Addictive Behavior, University S. Anna Hospital and Health Trust, Ferrara, Italy; k Centre for Addiction and Mental Health, Department of Psychiatry, University of Toronto, Toronto, Ontario, Canada; l University College London Great Ormond Street Institute of Child Health, University College London, London, United Kingdom; m Department of Psychology, Durham University, Durham, United Kingdom; n Dutch Society for Gender & Health, the Netherlands; o Gendered Innovations at European Commission, Brussels, Belgium; p Laboratory for Research in Neuroimaging, Centre for Research in Neurosciences, Department of Clinical Neurosciences, Lausanne University Hospital and University of Lausanne, Lausanne, Switzerland; q Department of Psychology, University of Oslo, Oslo, Norway; r Department of Psychiatry, University of Oxford, Oxford, United Kingdom; s Department of Child and Adolescent Psychiatry, Center of Expertise on Gender Dysphoria, Amsterdam University Medical Center, Vrije Universiteit Amsterdam, Amsterdam, the Netherlands; t Department of Clinical Psychology, Leiden University, Leiden, the Netherlands

**Keywords:** Brain, Gender, Mental health, Neurodiverse conditions, Sex

## Abstract

There are prominent sex/gender differences in the prevalence, expression, and life span course of mental health and neurodiverse conditions. However, the underlying sex- and gender-related mechanisms and their interactions are still not fully understood. This lack of knowledge has harmful consequences for those with mental health problems. Therefore, we set up a cocreation session in a 1-week workshop with a multidisciplinary team of 25 researchers, clinicians, and policy makers to identify the main barriers in sex and gender research in the neuroscience of mental health. Based on this work, here we provide recommendations for methodologies, translational research, and stakeholder involvement. These include guidelines for recording, reporting, analysis beyond binary groups, and open science. Improved understanding of sex- and gender-related mechanisms in neuroscience may benefit public health because this is an important step toward precision medicine and may function as an archetype for studying diversity.

## In This Issue

There are significant differences between males and females in the ways that mental health issues like autism are expressed and diagnosed, but we don't fully understand why these differences exist. This lack of understanding is harmful for people with mental health problems. To address this, a team of scientists, psychiatrists, psychologists, and policymakers worked together for a week to find out what is blocking progress in studying these differences. They came up with suggestions for how to improve research methods, involve the public and patients, and share information openly. These recommendations could help us better understand these differences and improve mental health care for everyone.

SEE COMMENTARY NO. 100292

## Background

**Figure 1:**
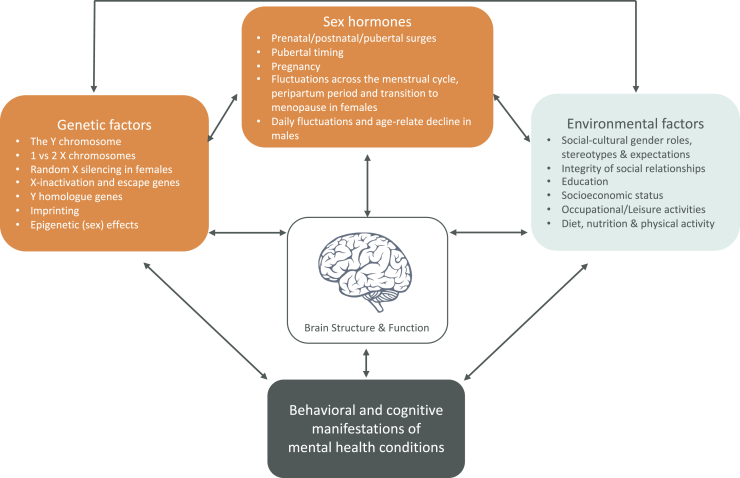
Both sex-related attributes (orange) and gender-related social-cultural factors (blue) moderate and mediate differences in the emergence, expression and diagnosis of mental health conditions. Moreover, sex- and gender-related effects interact and converge mechanisms shaping neurodiverse conditions. They moderate and mediate the behavioral and cognitive manifestations of neurodevelopmental health conditions and shape brain development. In addition, sex differences in the brain may act as compensating mechanisms that will limit sex differences in behavioral and cognitive outcome patterns (e.g., X-inactivation). It is important to better understand these differential pathways and how they interact to reduce recognition and diagnostic biases.

There are prominent sex differences in the prevalence of numerous mental health and neurodiverse conditions (1). For example, females are twice as likely as males to be diagnosed with depression and anxiety disorders, and males are more often diagnosed with attention-deficit/hyperactivity disorder and autism spectrum disorder (2, 3, 4, 5, 6). Moreover, there are sex differences in the expression of symptoms. Sex differences in mental health may partially reflect differential biological susceptibility expressed within the brain. Note that we refer to sex as a biological status (male, female, or intersex). However, differences between males and females may also stem from indirect pathways including sociocultural gender-related expectations that interact with biological factors and behavioral adaptations (e.g., camouflaging autism in women) (7). Importantly, both sex- and gender-related factors also differentially affect the expression and life span course of mental health conditions (8) (Figure 1). However, one of the largest challenges is that sex and sociocultural gender-related mechanisms are particularly complex to disentangle and cannot be captured by animal models. Therefore, in this paper, we focus primarily on research involving human participants. Challenges in this research field have been raised by others, along with recommendations for future research (9, 10, 11, 12, 13, 14, 15, 16, 17, 18, 19, 20). However, despite these recommendations and initiatives, many studies conducted to date have not examined potential sex- and gender-related mechanisms in the neuroscience of mental health (21). Moreover, results have often been misreported or interpreted in oversimplified ways (22). This contributes to stereotyping and may have harmful consequences for the diagnosis and treatment of mental health conditions. Improving our understanding of how sex and gender impact the brain and mental health throughout life is vital for public health.

In the current paper, we discuss what the main barriers are that withhold researchers from addressing and understanding sex- and gender-related mechanisms^1^. We identify what we believe are central problems in this research field and give recommendations for future studies. Most recommendations have been put forward by others, and this paper consolidates them. This work is the product of a collaborative 5-day workshop, with researchers, clinicians, and policy makers, that took place at the Lorentz Institute at Leiden University in November 2022. We acknowledge that our group has a majority White ethnic cisgender background, and we explored this topic from a mainly European perspective. Below, we first outline and discuss the current state of sex and gender research in the neuroscience of mental health. Second, we provide recommendations aimed at achieving a better understanding of the roles of sex and gender in the neuroscience of mental health.

## The Current State of the Field

### Biases

The biomedical field has a long history of including mostly male individuals in both animal studies and human preclinical and clinical trials. Ten years ago, only 15% of studies included both male and female participants (23). Since 1993, the U.S. National Institutes of Health has required the inclusion of females in clinical trials (NOT-OD-15-102) but has only required the inclusion of sex as a biological variable since 2016 (24). Since then, several other government funding agencies have recommended studying both males and females. This has led to a 50% increase in the number of neuroscientific research papers that include males and females (21,25). Nevertheless, the number of studies that have accurately reported or statistically addressed sex or gender as a factor in their analyses has not increased: less than half of biomedical studies have made reference to sex-related mechanisms (26,27). Within the neuroscientific field, males and females have been compared in only 5% of studies and emphasized even less often in the interpretation of results (15,21,28). Moreover, many studies that have made claims about sex- or gender-related mechanisms have not statistically tested them directly (21,25,26,28). Not only can this lead to misinterpretation of sex and gender differences, but these issues have also stymied the understanding of potential sex- and gender-related mechanisms in neurobiological vulnerability to mental health problems.

### Origins of Biases

Historical, societal, methodological, and biological factors have historically led to the predominant focus on male participants in medical research. Patriarchal norms excluded women from these fields, perpetuating their underrepresentation in scientific studies and clinical trials. Gender norms and inequality also influenced health disparities, with female-specific issues often being overlooked (12,29). The historical bias toward the male body as the norm continues to affect our understanding of sex and gender mechanisms in mental health neuroscience.

Methodological and biological concerns have included fertility-related health risks (30) and the beliefs that females are more variable than males (31) and that the female menstrual sex hormone fluctuations will reduce statistical power. However, meta-analyses in rodents have shown that there is no statistical difference between sexes in the variability of a variety of physiological and behavioral factors (32,33). At the same time, the differences in sex hormone fluctuations underlines the importance of including males and females in research designs because, for example, pharmaceutical drug-related health risks may impact males and females differently (34). Furthermore, a number of measurement tools are male-oriented (35, 36, 37). In addition, there is the misconception that including sex as an additional independent variable in analyses could potentially reduce statistical power. However, it should be noted that this does not always apply (38, 39, 40). These biases and misconceptions persist, hindering the translation of greater female inclusion in neuroscience studies and a deeper understanding of sex differences in brain and behavior.

## Recommendations

There are numerous barriers and challenges in neuroscience regarding understanding sex and gender differences in mental health. We identify 3 key domains: research methods, diagnosis/treatment, and stakeholder collaborations, each with unique challenges. Below, we outline these challenges and offer recommendations. Regarding research methods, we address procedures for recording, reporting, and analyzing sex/gender in mental health neuroscience studies. In the context of diagnosis/treatment, we explore the clinical advantages of studying brain and mental health sex/gender differences. Finally, we discuss obstacles in stakeholder collaborations.

### Research Methods: Recording

#### Conflated Terminology

Accurately recording, reporting, and analyzing sex and gender in neuroscience research on mental health is important, but it can be challenging for several reasons. First, terminology is frequently conflated because sex and gender are often incorrectly used interchangeably in the scientific literature (41). Accordingly, we recommend that research reports explicitly state whether sex or gender (or both) are the variables of interest and specify in detail how they were recorded. Moreover, the term gender is multifaceted and has diverse definitions, emphasizing the need for a clear specification of the concept of interest. Two aspects that are most often (intended to be) studied in neuroscientific research are gender identity [i.e., individual level, e.g., woman, man, nonbinary, gender fluid, transgender, and many more (42,43)] and societal gender expectations for roles and behaviors (i.e., societal level). These constructs are different and uncorrelated, each having their own dimensionalities and conceptualization. Moreover, they are driven by, and may affect, very different mechanisms in the brain and behavior. If one is interested in gender identity, including a standardized question on gender identity is recommended (Table S1) (20,44). We encourage inclusion of these questions because it will also increase the number of datasets available for exploring the effects of gender identity. Recording sociocultural gender-related mechanisms requires a very different approach. There is no gold standard for doing so because there are many different facets that vary culturally. Examples of recordings or manipulations of sociocultural gender constructs are gender stereotypes, gender beliefs, stereotype susceptibility, or cross-cultural comparisons (20,45, 46, 47, 48).

#### Nonbinary Sex Differences

In addition, although gender identity is often viewed as a categorical or dimensional measure, biological sex and sociocultural gender constructs are frequently recorded and analyzed in binary terms. However, the downstream effects of these constructs may very well encompass nonbinary end points. For example, although relatively rare, intersex variations can lead to chromosome configurations other than XX and XY. These variations may be overlooked when sex is recorded at birth. Another example of nonbinary sex-related mechanisms is differences in X-chromosome expression (X-inactivation in females) rates between the sexes (49). This results in a dosage or distribution difference for these genes rather than a strict binary sex difference. Furthermore, gonadal hormones also do not show binary differences but rather level differences between the sexes. These differences can be amplified or attenuated by sex-specific temporal fluctuations. For example, testosterone levels in males show daily variations, peaking around 10 am and declining thereafter (50). In contrast, females show hormone level fluctuations throughout puberty, the natural menstrual cycle, the peripartum period, and menopause (1,51, 52, 53, 54). Likewise, environmental interactions (related to sociocultural gender differences) also fluctuate across the life span, such as caretaking duties and career development (12). Research reports often fail to record these relevant sex-/gender-related variables.

We recommend carefully assessing what measures of sex and gender are required to match the research question of interest. In addition, we recommend using recording standards to make research data interpretable and comparable across studies (see Table S1) (44). This recording standard includes a 2-step recording of sex in which both sex recorded at birth and current sex are assessed. This will minimize the number of missed intersex conditions and has the additional value of allowing the pooling of data from different studies, thereby increasing sample sizes for intersex conditions that are currently scarce.

#### Sex and Gender Interact

Perhaps the most challenging aspect of recording sex and gender is that they vary under different conditions and that sex and gender mechanisms may interact (20,55,56). For example, in animal studies, different conditions for males and females (e.g., housing in individual or group cages) may mediate the variable of interest (e.g., stress response) (11). This not only limits the generalizability of sex-related findings but is also particularly complex in human studies because sex is highly correlated with sociocultural gender differences (18). Research designs cannot always distinguish between sex- and gender-related factors (e.g., most females are exposed to gender expectations of women). Thus, any observed sex differences may be mediated by an underlying unknown set of variables. We recommend that when the effects of sex and gender cannot be explicitly distinguished, the term sex- and/or gender-related mechanisms should be used. Furthermore, a biopsychosocial approach could be used to unravel the mediating and moderating roles of sex and gender on the brain and behavior. This can be seen, for example, in the study by Hausmann *et al.* (47), where results showed that sex hormones mediated the effects of gender stereotypes on performance on a mental rotation task.

Moreover, not only may there be a mediating role of sex-related factors, but sex-related mechanisms can also moderate the effects of gender on the brain and behavior. These mediating and moderating effects particularly complicate observational study designs and the understanding of sex- and gender-related mechanisms in neuroimaging research in humans (19). An example of a sex difference that may act independently of gender is Rett syndrome. This is a severe neurodevelopmental disorder caused by a mutation on the methyl-CpG-binding protein 2 gene, *MECP2*, on the X-chromosome. This syndrome is nearly completely absent in males because the mutation has lethal effects. However, most sex- and gender-related mechanisms interact. This is important to take into account to better understand how individual factors (e.g., genetics, brain, hormones, cognition) bidirectionally interact with social factors at the level of the family, peers, and school, which in turn are affected by culture and our society and directed by, for example, policy (57, 58, 59, 60). In a biopsychosocial model, biological mechanisms (e.g., sex hormones) and environmental factors (e.g., sociocultural gender expectations) are not treated as independent variables, but rather as continuous and inseparable variables (61,62).

### Research Methods: Reporting and Analysis

In addition to the importance of accurately recording sex and gender, there are specific challenges related to reporting and analysis. Even though there has been a large increase in studies that include both males and females, over 90% of studies in the neuroscience field do not report or statistically compare the sexes (11,15,21,28). This is a missed opportunity. Even if sex and gender are not the topic of interest, reporting on sex and gender results could potentially boost our understanding. Therefore, we recommend the standardized reporting of demographic characteristics of samples. This would enhance transparency, enabling accurate interpretation of results and facilitating data extraction, such as in meta-analyses. We recommend including a minimum number for each sex and/or gender within the experimental groups together with a demographics table stratified by sex. The field could capitalize on open science advances by encouraging researchers to share their data, methods, and results. One barrier that impedes researchers from doing so is the fact that these open science practices require training and can be time-consuming. Not only should training on open science practices be provided to scientists, but such initiatives could also be recognized by policy makers and university guidelines to reward scientists for their efforts.

Beyond the reporting of sex and gender information on samples, there are a number of challenges in analyzing sex- and gender-related mechanisms that require moving beyond traditional statistical methods. As mentioned above, both sex- and gender-related mechanisms may not result in binary outcomes in the brain and behavior. For example, when similarity analysis is used rather than classification analysis, for example, brain structures do not map onto 2 sex-typical profiles after overall brain size has been taken into account (63). In addition, for a number of characteristics, including brain structure, males have shown greater between-person variability than females (64, 65, 66, 67, 68, 69, 70). For other traits, females have shown greater within-person variability than males, for example, where sex and/or gender differences were masked by menstrual cycle day (71). The (nonbinary) interpretation of sex differences in human brain structure is further complicated by the finding that brains consist of a mixture of male and female features, resulting in mosaic patterns that have been shown to be highly individualized (72). Therefore, traditional (binary) group analyses may lead to overestimations or misinterpretations of sex/gender differences. Analysis and interpretation of sex- and gender-related mechanisms are also complicated by their interactions, as well as by how their interactions can change over the course of the life span (e.g., prenatal and pubertal hormone exposure shapes brain structure and function, and during adolescence there is a shift in societal expectations that is different for boys and girls). Therefore, longitudinal study designs are warranted to better understand how sex and gender mechanisms interact across different developmental stages.

We recommend contextualizing group differences by comparing sex/gender differences observed in brain structure to sex differences observed in body size or other explanatory variables (such as age or socioeconomic status). In addition, it is equally important to report when no significant group differences are observed, particularly for meta-analyses. Equivalence testing could also be applied to determine whether null findings are explained by sample size or the absence of group differences (73).

### Diagnosis and Treatment

The urgency to advance our knowledge of sex- and gender-related aspects of mental health is accentuated by the significant increase in the number of children, adolescents, and adults with mental health problems and who have been diagnosed with a mental health condition over the last 2 decades (74, 75, 76, 77). Across societies and cultures, mental health services are overwhelmed with referrals, and initial cohort studies have confirmed these clinical observations (78, 79, 80). Importantly, these tendencies are sex-/gender-specific and differ across the life span (2).

#### Gap Between Neuroscience and Clinical Practice

One major challenge is that there is no one-to-one mapping of fundamental insights from neuroscience to daily clinical practice. In clinical practice, a holistic approach is ideally used to interpret, explain, and treat the problems reported by a patient. However, such a holistic approach has rarely been applied in neuroscientific studies, where small pieces of the large puzzle are tackled to increase our theoretical understanding of the interaction between the brain and mental health.

Even though bridging the gap between research and the clinic is made challenging by many barriers, we strongly recommend making every effort to do so. A first step would be to involve stakeholders such as patients, families, and clinicians in the research cycle to a larger extent. This will facilitate the translation of complex theoretical models into actionable steps for clinical practice. However, we recognize that the implementation of basic neuroscientific findings in daily practice requires different research phases/areas and involves multiple steps from fundamental knowledge (e.g., at the cellular level) to clinical application (e.g., randomized controlled trials and implementation studies). To ensure the success of this translation process, communication between these different levels of research in collaboration with patients and their clinicians is key. The different perspectives will not necessarily align because these perspectives are multidisciplinary and multidimensional in nature. Accordingly, they represent different backgrounds (e.g., educational, cultural, or socioeconomic backgrounds) related to different fields of daily practice. Taking different perspectives of community members and patients, the clinical perspectives of mental health care providers, and the perspectives of (neuro)scientists into account requires time, effort, and financial compensation during different parts of the research cycle.

#### Group-Based Modeling

A second challenge is that group-based models and binary approaches are limited. These include the classical case-control or male–female study design; such binary approaches do not capture the full complexity of underlying populations. Moreover, the traditional case-control paradigm is focused on classification of mental health disorders by DSM-5 and ICD-11 and thereby ignores the heterogeneity and high co-occurrence of mental health conditions. Despite the value of studies that use group-based paradigms, there is growing consensus in the field about the need to include alternative research approaches that cut across the traditional categorical diagnostic boundaries, including transdiagnostic and dimensional frameworks (81, 82, 83)^2^.

We propose several recommendations to go beyond group-based research. One recommendation is to capitalize on the advantages of big data. Analyzing big open datasets can reveal previously unnoticed associations and allow researchers to study variations and trends across populations and time, as well as make it easier to validate and replicate results and allow for application of advanced computational and statistical methods. This will allow the field to move beyond the classical case-control paradigm and increase our understanding of mental health using transdiagnostic and dimensional approaches. Examples of these approaches are network analysis of psychopathology [see for example (84,86)] and normative modeling to capture the heterogeneity of mental health problems (87,88). A subsequent action to consider involves incorporating aspects of sex/gender into these advanced methods. However, while these advances add to our scientific knowledge, at the same time, large datasets may not capture details and nuances related to aspects of sex, gender, and mental health. Moreover, big datasets may contain biases related to participants or collection methods, and handling large and complex datasets can be challenging and require training. In addition, with large datasets and application of complex models, there is a risk of overfitting. Therefore, we recommend combining these large-scale studies with smaller and specifically designed studies that focus on complex and nuanced aspects of sex- and gender-related factors in the neuroscience of mental health. Additionally, we suggest further integration of qualitative and quantitative research designs; these mixed-method approaches combine the advantages of both research methods, capture nuances at an individual level, and have the advantage of generalizability to group levels.

### Stakeholder Collaborations

In the diagnosis and treatment section, we already highlighted that bidirectional communication between scientists and societal stakeholders is an important step toward better understanding of sex- and gender-related factors in the neuroscience of mental health. In this section, we will further elaborate on the significance, potential barriers, and action points to facilitate collaborations between academia and stakeholders. Below, we focus on science communication and stakeholder involvement. We outline the barriers that hinder researchers from engaging in science communication and including stakeholders in discussions on the topic of sex- and gender-related mechanisms in neuroscience, especially concerning mental health. Our recommendations are provided to mitigate these challenges.

#### A Sensitive Societal Matter

One potential barrier for researchers to address sex and gender is that this is a socially sensitive subject. The topic is subject to societal opinions, which often evokes controversy and misunderstanding. Researchers have raised the issue that this factor hinders them from effectively communicating about or sometimes even studying this topic. A way to foster knowledge transfer and dissemination on this sensitive topic could be including training on science communication and stakeholder engagement practices from school and undergraduate degrees onwards. However, training of such a skill set is not part of most academic curricula and is only accessible to students, trainees, and researchers on a limited basis. Despite this, these practices enable the addressing of various United Nations Sustainable Development Goals related to sex and gender equality and equity in well-being and health. The absence of good practices and training on stakeholder collaborations may have negative consequences. For example, stakeholder collaborations may devolve into tokenism where participation is used to provide a false impression of engagement (89,90), consequently eroding the public trust of stakeholders in collaboration with researchers. Therefore, training is of key importance and requires adequate funding to allow for collaborative research.

#### Recognition and Reward

Science communication and stakeholder involvement are both time-consuming activities. Engaging in these activities will restrain researchers from conducting traditional scientific activities. Currently, however, the efforts of researchers are undervalued and hardly recognized or rewarded within academic, professional, or funding institutions. Therefore, academic institutions should recognize and reward the investments and outputs of researchers who engage in social responsibility practices to a larger extent. In addition, trained support staff could help researchers engage in these activities and thereby increase the impact of their scientific work. We strongly recommend that academic, educational, and communications institutions seek to employ specialists in science communication and public engagement. Furthermore, fostering collaborations with professional organizations can help facilitate dedicated science communication and media training. Such training should encompass researchers, educators, and journalists, ultimately enabling them to interpret sex and gender findings more effectively. Such efforts will contribute to enhancing the broader impact of research in this field.

#### A Facilitating Role for Funding Agencies

Funding agencies should facilitate sex and gender research because such research will not be facilitated without resources. We have some specific recommendations for investments in stakeholder engagement and science communication practices that could also be expanded within existing structures. As mentioned above, several funding agencies now set requirements for inclusive research across sex and gender factors. This concept could be expanded upon, with funding agencies playing a more active role in providing substantial grants. These grants could enable the establishment of collaborative initiatives that promote stakeholder engagement throughout different aspects of the research cycle. For example, funding agencies could facilitate cocreation with community members and patients to define research questions that reflect societal needs to allow maximum impact of the research on the communities or to support skillful science communication that will continue to show an impact throughout the funded project. Furthermore, we recommend that professional bodies, regulators, and funding bodies invest in the infrastructure that allows researchers to share guidelines and report on sex and gender measures, ultimately facilitating the application of research outcomes on sex- and gender-specific support pathways in mental health. Lastly, many of the aforementioned resources (training, guidelines, materials) could easily be made publicly available and promoted via existing open science platforms. We would like to highlight that improving equity and equality in mental health outcomes via neuroscience research requires interdisciplinary work and collaborative effort in which all stakeholders have an important role to play.

## Summary

Sex- and gender-related mechanisms have long been ignored. This is in part related to the complexity of their interactions. Furthermore, sex and gender interactions vary over the course of the life span. Moreover, the holistic approach to understanding symptom expression in a single patient does not translate into neuroscientific research practices and vice versa. Our recommendations to move this field forward include using explicit descriptions of sex and gender constructs in a research article, improving sex and gender recordings, including underrepresented groups in the research process, and accurately reporting and analyzing sex- and gender-related mechanisms and their interactions. To do so, interdisciplinary collaborations, which require both time and financial investments, are key.

There is a need for a call for action to develop and deliver more sex- and gender-sensitive treatment programs across the globe (91, 92, 93) as a first step in personalized treatment programs (94). We believe that the recommendations put forward in the current paper will advance the field and increase our understanding of sex- and gender-related mechanisms in the brain and behavior and their relationships to mental health and beyond to better address diversity.

## Footnotes

1 The construct “sex” refers to related but separable biological constructs. These include genetic components, hormones, and cellular differences. The construct “gender” is an umbrella term that includes gender identity and sociorelational constructs that range from individual to societal attributes. From research conducted to date, it is unclear how sex and gender interact; therefore, we sometimes refer to sex and gender specifically and at other times acknowledge that both constructs are involved. This was a deliberate decision because we acknowledge that sex and gender have separate as well as interactive and indistinguishable impacts.

2 This perspective paper focuses on sex- and gender-related aspects of the neuroscience of mental health. The Lorentz workshop and this perspective paper were not designed to define mental health, but we conceptualize it as a societal problem. We refer to perspective papers on challenges related to defining mental health [e.g., Borsboom (84), Cuthbert and Insel (81), and Kendler *et al.* (85)].
