# Supplementary material for: Recommendations for a Better Understanding of Sex and Gender in the Neuroscience of Mental Health
Source: Biol Psychiatry Glob Open Sci. 2023 Dec 30;4(2):100283. doi: 10.1016/j.bpsgos.2023.100283 (PMC10837069; doi:10.1016/j.bpsgos.2023.100283)
Supplement: Table S1 [file mmc1.pdf]

SUPPLEMENTARY INFORMATION

Recommendations for a Better Understanding of Sex and Gender in Neuroscience of Mental Health

Wierenga *et al.*

Supplementary box 1. *Recommended recording standard on sex and gender identity*

We recommend to include the following three questions to the recording standards to assess biological sex and gender identity:

What is your sex recorded at birth?

I was assigned female at birth

I was assigned male at birth

I was born Intersex and was assigned female at birth

I was born Intersex and was assigned male at birth

I was born Intersex and my sex is not entirely clear

What is your current recorded sex?

Female

Male

Intersex ___________

What is your current gender identity?

Woman

Man

Non-binary

Prefer to self-describe: _____________

BOX 2. Barriers and Recommendations for Human Neuroscientific Research on Sex and Gender in Mental Health

Barriers	Recommendations
**Research practices and methods - recording**	
'Sex' and 'gender' terminologies are often conflated in scientific literature	Explain 'sex' and 'gender' terms in academic papers (e.g. use sex/gender if mechanisms are unknown)
Sex and gender related factors are not binary (e.g. intersex, gender identity)	Use standardized recording of sex and gender identity
	Use stakeholder engagement in research cycle to keep constructs and measures up to date
The term 'gender' is multifaceted	Be clear about what construct you are refering to (e.g. gender identity or social cultural gender effects)
**Research practices and methods - Analysis**	
Sex and gender interact	Implement the biopsychosocial model
Sex and gender interactions vary across the lifespan	Interdisciplinary collaborative efforts are key to solve differential pathways of sex and gender and how they interact
	Collaborative multidisciplinary efforts are time consuming and funding is needed
Sex (and gender) related endpoints are not always binary	This requires the implementation of non-binary analysis (e.g. data driven methods, or variability comparisons)
**Diagnosis and treatment**	
Applying scientific knowledge in clinical practice is currently suboptimal	Involve stakeholders in all phases of the research cycle to improve translationality
Highly focused research questions do not line up with a holistic approach warranted when helping patients	Integrate qualitative and quantitative research designs
Group-based models and binary approaches do not fully capture neurodiversity and non-binary seks or gender related endpoints	Apply novel anlaysis methods that capture variability
	Capitalize on the advantages of big data to move beyond the classical case-control paradigm
**Stakeholder collaborations**	
The societal sensitivity of sex and gender research may withhold researchers from science communication	Involve stakeholders with various backgrounds at different levels of research design to e.g. discuss how to best communicate findings
There is a lack of access to training science communication and stakeholder engagement practices	Develop training programs and implement this in curriculi
Open science practices and stakeholder collaborations are currently not rewarded and only to a limited extent funded	Reward and recognize open science initiatives of individual researchers and provide funding opportunities for science communication, open science and stakeholder engagement practices
